# Glucans, Paramylon and Other Algae Bioactive Molecules

**DOI:** 10.3390/ijms24065844

**Published:** 2023-03-19

**Authors:** Laura Barsanti, Paolo Gualtieri

**Affiliations:** Istituto di Biofisica, Area della Ricerca CNR, via Moruzzi 1, 56124 Pisa, Italy

Algae (macro- and micro-algae) can be defined as light-driven cell factories that synthesize bioactive compounds consisting of primary metabolites (i.e., molecules that play a direct role in the algae metabolism) such as lipids, amino acids and carbohydrates, and specialized secondary metabolites, such as pigments, sterols and vitamins. Many of these compounds are characterized by bioactivity, i.e., they can achieve a defined biological effect on a target, which can be both health-promoting and disease-suppressing. Humans and other higher organisms are unable to synthetize most of these molecules or synthetize them in a too low amount, and external administration in the diet is strongly recommended to avoid dangerous deficit. Hence, safe dietary supplements are important for long-term physiological health benefits; this is a role that can be played by algae as dietary supplements in food, and as therapeutic adjuvants for the management of many different diseases. The benefits algae can provide range from dietary benefits, decreasing the risk of cardiovascular diseases, cancers and diabetes, to health benefits, promoting a better quality of life, since, from a therapeutic point of view, they possess antioxidant, antimicrobial, antitumor and immunomodulatory characteristics [1].

The properties and benefits of algal compounds with nutraceutical, pharmacological, and biomedical interest [2,3] are the main topics of this Special Issue in *International Journal of Molecular Sciences*, with major emphasis on polysaccharides, of which are the most exploited compounds worldwide. This main topic is expanded to include polysaccharides of other origins, such as fungi [4,5], which also possess biological activity, and review the defense mechanisms triggered by the interaction of these compounds with organisms, which are at the base of immunity [6]. 

Barsanti et al. [2] described how establishing algae as an alternative supplement would complement the sustainable and environmental requirements in the framework of human health and well-being. The authors describe the proprieties and uses of the main micro- and macroalgae metabolites, their potential for application in the different industrial sectors, from food/feed to chemical and pharmacological, and the current technologies involved in bioactive molecule extraction strategies. 

Algae are one of the best approaches to address the surge in demand for healthier, more natural, sustainable products to bio-fortify food, to boost immune systems without the need of drugs, to replace synthetic antibiotics with novel compounds already present in nature and to have the same or higher efficacy against antibiotic resistance pathogens. Algal compounds can address the nutritional deficiencies of many foods and feeds and are a promising alternative to animal sources for vegetarian and vegan consumers. However, although the numbers of algal bioactive molecules are commercially available and tested, the social acceptance of health benefits is still uncertain. 

Macroalgae present advantages over microalgae due to the lower cost of cultivation—especially in the offshore set, which prevents the utilization of land and does not compete with agriculture crops—and to the high amount of achievable biomass. Additional nutrients are not generally needed; the carbon footprint is negligible and is limited to the biomass-processing steps, as well as the risk of the eutrophication of aquatic systems, making macroalgae production an environmentally friendly exploitation. Moreover, due to their high capacity of carbon fixation, macroalgae have the potential to act as bioremediators of euthrophic coastal waters. 

Microalgae present advantages over macroalgae due to the abundance of bioactive compounds already tested in many years of research and investigation. The main applications of microalgae have always been (and still are) in aquaculture as feed for the growth of larvae and juvenile shellfish and finfish in order to increase their nutritional value and improve survival and immune defenses. These effects are also increasingly exploited for human health. In the clinical daily routine, the most important expected progress in this field would be an increased use of algal polysaccharides such as alginate gels for wound healing and for antiulcer agents and β-glucans (paramylon nanofibers) to be immunostimulants. These molecules, as all the other bioactive compounds of algal origin, would be best utilized when novel extraction and purification methodologies are developed to allow for a sustainable cost-effective industrial utilization [7]. So far, the limitations of developing industrial algae biotechnology are mainly represented by the high downstream process and bio refinery costs of the biomass, which can account for up to 50–80% of the whole amount. Because of these limitations, research efforts should focus on advancing farming methodologies, new biomolecules identification and characterization, high yield extraction methods and low energy consumption processes.

Barsanti et al. [2] list some of the bioactive compounds present in micro- and macroalgae, such as pigments, polyunsatured fatty acid (PUFA), polyphenols, polysaccharides and vitamins. Since the major bottleneck in the exploitation of microalgal biomass for the production of these high-value compounds is the low productivity of the culture, both in terms of biomass and product concentration, detailed data on the specific product extracted from specific algae, and on their cultivation and methods of extraction of the bioactive molecules, are given together with economic data of the different categories of compounds. 

The algal global world market value is nowadays approximately USD 6 billion, with the global production of algae dominated by marine macroalgae, grown in both marine and brackish waters; cultivation has overshadowed the production of algae collected from the wild, which accounted for only 3% of total production in 2019. Among the different algal compounds, pigments had an overall market value of USD 115 million in 2018, which is expected to double in 2028 due to their high commercial value as natural colorants in the nutraceutical, cosmetic and pharmaceutical industries, in clinical research and molecular biology and as natural dyes in the textile industries; these pigments have a wholesale price averaging USD 15.000 t^−1^. 

PUFAs are gaining increasing importance as valuable pharmaceutical products and food supplements due to their beneficial effect on human health. Due to the presence of specific desaturase enzymes, algae are essentially the only organisms able to produce long-chain PUFAs, from 14 to 24 carbons. Humans and other animals cannot convert ALA to EPA and DHA at the required levels, so dietary additions of these essential fatty acids are critically important for their health. Microalgae are the primary producers of EPA and DHA that are eventually accumulated through the various trophic levels. Changes in microalgal lipid content are carried on up the food chain, impacting the growth and dietary make-up of zooplankton, crustacean larvae, mollusk and some fish. This subsequently affects the accumulation of EPA and DHA fatty acids in higher organisms and humans. Consequently, lipid profiles in microalgae play a vital role in maintaining the integrity of the world’s aquatic food webs. The selling price for PUFAs sold as micronutrients and anti-inflammatory compounds can be higher than USD 5 million t^−1^.

Most of the polysaccharides synthesized by algae are not digested by humans, and therefore, can be defined as dietary fiber, i.e., physiologically beneficial non-digestible carbohydrates [8]. Dietary fiber included in algae is classified into insoluble and water-soluble fiber. Soluble fiber is characterized by its ability to form viscous gels, in contact with water, in the intestinal tract; it is fermented in high proportions, and its main properties are related to the decrease in cholesterol and glucose in blood and the development of intestinal microbiota. Insoluble fiber does not form gels in contact with water but is capable of retaining water within its structural matrix, producing an increase in fecal mass that accelerates intestinal transit with a marked laxative and intestinal regulating effect. Moreover, sulfated polysaccharides act as a coating material on the surface of sanitary items to prevent infection from epidemic diseases such as COVID-19 [9].

Among polysaccharides, β-glucans are the most important, and are present inside the algae as storage compounds or wall components. β-glucans have a potent immunomodulating activity [10,11,12,13,14]. Their action is mediated through receptors such as Dectin-1 (a C-type lectin receptor), Toll-like receptors, complement receptor 3, scavenger receptor and lactosylceramide. β-glucans function as pathogen-associated molecular patterns (PAMPs) and can be non-specifically recognized by pattern recognition receptors (PRRs) present on the surface of the innate immune system cells. The selling price of β-glucans can be more than USD 0.5 million t^−1^.

The vitamin profile of algae can vary according to algal species, season, growth stage and environmental factors. Algae can be considered a functional source of these essential compounds to fulfill the dietary requirements of humans and animals as food or feed complements. Vitamins from algae are not purchased as single biomolecules but as part of other algal supplements (food and nutraceutical); hence, their market value is not possible to assess.

Comer et al. [3] tested two 5-mer oligoglucans (β-1,3 laminaripentaose, and α-1,4 maltopentaose) for their capacity to induce cytokine expression. They verified an immune-stimulant effect of β-1,3 oligoglucans on T lymphocytes, with a significant increase in the mRNA levels of T-cell-activation-associated cytokines, especially in the presence of the agonistic anti-CD3 antibody and the absence of dectin-1. The authors used a model cell type that does not express dectin-1 so that any observed activity should be due to a mechanism not involving this receptor. The binding of β-1,3 oligoglucans was seen to be selective because the affinity of α-1,4 oligoglucan for the same site was much lower. The affinity of β-1,3 oligoglucans for CD28 was similar to that for dectin-1, which is well-recognized as an innate immune system receptor for β-1,3 glucans. The authors suggest that β-1,3 oligoglucans can promote this stimulation by binding with clusters of differentiation, such the CD28, which was previously unknown. To achieve this result, they performed a series of molecular dynamic simulations and free-energy calculations to verify the interaction of β-1,3 glucans with CD3, and CD28 potential immune antigene receptors, expressed in T cells. These simulations reveal little association between β-1,3 oligoglucans and CD3, and a strong one with a specific binding to CD28 near the region identified as the binding site for its natural ligands CD80 and CD86. The free-energy calculations showed that the dissociation constant for binding to this site on CD28 is in the low millimolar range [15]. They conclude that their results strongly suggest that the binding of β-1,3 oligoglucans to CD28 present on the T-cell plasma membrane functionally stimulates T-cell activation in collaboration with CD3 activation, without using a dectin-1-mediated mechanism. Their work provides evidence that CD28 plays a role in the immunostimulatory effects of β glucans, which may be relevant in the β glucan-induced anticancer immunity observed in preclinical studies. 

Anionic polysaccharides have attracted much attention in recent decades for their inhibitory activity against viruses [16,17,18]. Among them, enveloped viruses, such as herpes simplex (HSV-1 and HSV-2), influenza, HIV-1 and 2, dengue (DENV 1-4) and SARS-CoV-2 (COVID-19), infect mammalian cells, causing severe diseases and affecting millions of people all over the world. Drugs currently used to treat HSV-1 infection are acyclovir and its analogs, but the number of viral strains resistant to these drugs is increasing, and there is no specific treatment approved for dengue. Among anionic polysaccharides, the sulfated ones, extracted mainly from seaweed, stand out as showing potent activity as antiviral drugs, thus offering a promising alternative to synthetic drugs. They inhibit the first step of infection, where the glycoprotein on the viral envelope utilizes its positive charges to interact with negative charges of heparan sulfate (HS), one of the host cell surface receptors, which carries both carboxyl and sulfate groups. By inhibiting this step, sulfated polysaccharides mimic HS, thus blocking the virus from entering the host cell. Despite their biological potential against enveloped viruses, natural sulfated polysaccharides, mainly of seaweed origin, are only seasonally available, which limits their production throughout the year. Moreover, their purification from the carbohydrate extracts is a laborious process. The paper of Lopes et al. [4] explores the possibility to overcome these disadvantages by chemically derivatizing a (1,3)(1,6)-D-glucan-type polysaccharide, produced by the fungus *Botryosphaeria rhodina* MAMB-05 through a submerged fermentation process. This molecule possesses potent antiviral activity already tested against HSV and DENV [19]. To make botryosphaeran more similar to HS, the host cell receptor bearing both carboxyl and sulfonate groups, the authors used a botryosphaeran preparation containing both carboxymethylated and sulfonated derivatives. Carboxyl and sulfonate groups were introduced by carboxymethylation and sulfonylation reactions, respectively. Three derivatives with the same degree of carboxymethylation (0.9) and different degrees of sulfonation (0.1; 0.2; 0.4) were obtained. The derivatized compound was tested against herpes-simplex- (HSV-1 strains KOS and AR, sensitive and resistant to acyclovir, respectively) and DENV-2-enveloped viruses, and poliovirus, a non-enveloped virus. Carboxymethylated botryosphaeran did not inhibit the viruses, while all sulfonated-carboxymethylated derivatives were able to inhibit HSV-1 in a dose-dependent manner, demonstrating that the antiherpetic activity of native botryosphaeran was strongly enhanced by the derivatization procedure. Lopes et al. also demonstrated that DENV-2 was inhibited only by one of these derivatives with an intermediate degree of sulfonation (0.2), indicating that the dengue virus is more resistant to anionic β-D-glucans than the herpes simplex virus. By comparison with a previous study on the antiviral activity of sulfonated botryosphaerans, the authors observed that the presence of carboxymethyl groups might have a detrimental effect on antiviral activity, and that the inhibitory potency of the present derivatives was slightly weaker than that observed for sulfonated botryosphaerans reported in their previous work [19]. Therefore, mimicking heparan sulfate with sulfonated-carboxymethylated β-glucan derivatives did not result in a better degree of inhibition of HSV with respect to sulfonated β-glucan derivatives. 

Gorska-Jakubowska et al. [5] presented the results of their further research on the influence of selenium incorporation on the biosynthesis, structure and immunomodulatory and antioxidant activities of polysaccharides extracted from the fungus *Lentinula edodes*. 

Mushroom-derived polysaccharides, as algal polysaccharides, are active as antioxidants and have antitumor, immunomodulating, antibacterial, antimicrobial, antiviral, anti-obesity, hypolipidemic, antidiabetic and hepato-protective properties, among other activities [20]. Polysaccharides obtained from higher fungi affect different types of immune responses and are therefore collectively referred to as Biological Response Modifiers (BRM) [21]. 

Selenium, as an active center of enzymes involved in oxidative transformations, plays a key role in the resistance mechanisms of plant and animal organisms and possesses anti-tumor and immunomodulatory effects. The authors investigated whether the supplementation of the mushroom culture medium with selenium (in the form of sodium selenite) would lead to the incorporation of this element into the structure of the polysaccharides secreted into the medium by the mycelium of *L. edodes*. They isolated a selenium (Se)-containing high-1–6-branched exopolysaccharide, or cell-wall Se-polysaccharide, from *Lentinula edodes.* This compound was composed mainly of units of glucose, mannose, galactose and xylose, and bounded to a protein of molecular weight 4.5 × 10^3^ kDa. The structure of this carbohydrate-binding protein (15% protein in weight) was characterized by a significantly higher molecular weight with respect to a mammonoprotein previously isolated by the same research group. The X-ray absorption fine structure spectral analysis indicated that selenium in the Se-exopolysaccharide structure was present mainly in the IV oxidation state, while the simulation analysis in the EXAFS region suggested the presence of two oxygen atoms in the region surrounding the selenium. The authors demonstrated that selenium-enriched exopolysaccharides possess higher biological activity than the non-Se-enriched reference fraction, thus showing selective immunosuppressive activity, which is, however, significantly lower than that of the previously isolated mycelial polysaccharides [22]. The biological activity assays showed that the Se-enriched exopolysaccharide fraction significantly enhanced cell viability when incubated with human umbilical vein endothelial cells and protected them from oxidative stress; however, this effect was absent in the case of malignant human cervical cells. The extracted Se-exopolysaccharide also inhibited human T-lymphocyte proliferation induced by mitogens, without significant effects on B-lymphocytes. The authors suggest that the different mode of selenium binding and its higher degree of oxidation are responsible for the lower biological activity.

Immunity is the first line of defense in the integrity of the human (and vertebrate) body against foreign danger. It can be constitutive (natural or non-specific) and acquired (adaptive or specific); non-specific immunity is innate, while specific immunity needs time to become fully functional. Constitutive immunity recognizes foreign structures by PRRs, which evolve before the tissues, cells and effector mechanisms of adaptive immunity. Adaptive immunity gradually creates a more precisely targeted response to the “non-self” foreign antigenic structures that the individual encounters during its life. In addition, it stores, for its entire life, mechanisms of this specific response in the form of immunological memory [23].

The paper of Vetvicka et al. [6] deals with trained immunity, of which is one of the most interesting, potentially commercially and clinically relevant ideas of current immunology. The authors suggest that the binary classification of immune memory has become obsolete. They state, citing many experiments, that a less-specific trained immunity, realized by the epigenetic reprogramming of non-immunocompetent cells, may cross-protect against infectious agents; trained immunity is induced by a primary meeting with a pathogen and confers protection against a secondary infection independently on the mechanisms of adaptive immunity.

Vetvicka et al. introduced the hypothesis that innate immunity could be influenced by previous encounters with the PAMPs of pathogenic microorganisms, and they developed mechanisms to remember these structures. This ability of innate immunity to display a non-specific memory was coined “trained immunity” or “innate immune memory” by M. G. Netea et al. [24,25]. The authors described how trained immunity differs from the classical immunological memory of adaptive immunity, emphasizing the different rows of cellular populations and their different origins. They are primarily myeloid cells, monocytes and macrophages, NK cells and dendritic cells; even innate lymphoid cells (ILCs) are functionally different from those involved in classical immunological memory.

Originally, evidence for the existence of trained immunity in vertebrates was obtained from experiments with mice, which were protected against lethal bacterial infection with *Staphylococcus aureus* by non-specific substances, such as β-glucan [26]. The basic idea behind these findings was that certain challenges promoted a heightened response in myeloid cells upon subsequent infection with the same (and in some cases, different) pathogens. 

Further evidence that trained immunity was not dependent on the mechanisms of adaptive immunity, but the animals were protected against reinfection by macrophage activation and cytokine production, was demonstrated. The protective effects, which are not mediated by mechanisms of adaptive immunity but are realized mainly by macrophages, may also be induced by various pathogenic organisms, such as herpes virus-induced resistance against *Yersinia* and *Listeria*, bacteria, and the helminth parasite, *Nippostrongylus brasiliensis*.

To date, trained immunity has been studied mostly in teleosts, rodents and humans. An interesting hypothesis was raised by Quintin [27], who suggested that there are two immunologically opposite parts of the innate immunity memory—tolerance and trained immunity—and these parts might be epigenetically or mechanistically mirrored. Most studies have focused on changes in cells upon primary activation via numerous modulators, such as glucan. 

One of the most interesting studies focused on a theory that β-glucan-induced trained immunity can start antitumor activity. Prophylactic treatment with glucan caused lower tumor growth (which has been observed repeatedly in other studies), but the adaptive transfer of trained neutrophils into naïve animals suppressed cancer growth again. A detailed evaluation found a transcriptomic and epigenetic rewiring of neutrophils and entire granulopoiesis toward an anticancer phenotype. These findings might open a new window into cancer treatment [28].

The authors conclude that trained immunity can represent an evolutionary conserved phenomenon since its effects induced by microbial products such as β-glucans and lipopolysaccharides are accompanied by a more effective cytokine response, which could lead to an improved antiviral protection, even from the coronavirus disease, COVID-19 [29,30]; that trained immunity is realized by the epigenetic reprogramming of non-immunocompetent cells, primarily macrophages and NK cells, and is less specific than adaptive immunity, therefore offering cross-protection; and last but not least, that the various actions of trained innate immunity on precursor cells have a strong potential for therapeutic uses, particularly in infected and myelosuppressed individuals. 

All the papers present in this Special Issue highlight the potential of natural substances such as β-glucans with health-enhancing properties. No matter the source of these molecules, either algae or fungi, they exhibit a potentiating action on the immune system against microbes and toxic substances. Moreover, β-glucans are known to exhibit anticancer effects and can suppress cancer proliferation through immunomodulatory pathways. These molecules are biological response modifiers which can be used as a dietary supplement for their overall benefits, or are tailored to produce novel therapeutic approaches that are able to overcome the current obstacles in the treatment of many different pathologies. The main bottleneck to the full exploitation of these molecules is mainly represented by the high downstream and biorefinery processes that are necessary to guarantee a purified product, of which is needed to verify their actual efficacy in any future clinical trials.
